# Voice disorders and mental health in teachers: a cross-sectional nationwide study

**DOI:** 10.1186/1471-2458-9-370

**Published:** 2009-10-02

**Authors:** Eléna Nerrière, Marie-Noël Vercambre, Fabien Gilbert, Viviane Kovess-Masféty

**Affiliations:** 1 Foundation for Public Health, MGEN, Paris, France; 2 EA4069, Université Paris Descartes, Paris, France

## Abstract

**Background:**

Teachers, as professional voice users, are at particular risk of voice disorders. Among contributing factors, stress and psychological tension could play a role but epidemiological data on this problem are scarce. The aim of this study was to evaluate prevalence and cofactors of voice disorders among teachers in the French National Education system, with particular attention paid to the association between voice complaint and psychological status.

**Methods:**

The source data come from an epidemiological postal survey on physical and mental health conducted in a sample of 20,099 adults (in activity or retired) selected at random from the health plan records of the national education system. Overall response rate was 53%. Of the 10,288 respondents, 3,940 were teachers in activity currently giving classes to students. In the sample of those with complete data (n = 3,646), variables associated with voice disorders were investigated using logistic regression models. Studied variables referred to demographic characteristics, socio-professional environment, psychological distress, mental health disorders (DSM-IV), and sick leave.

**Results:**

One in two female teachers reported voice disorders (50.0%) compared to one in four males (26.0%). Those who reported voice disorders presented higher level of psychological distress. Sex- and age-adjusted odds ratios [95% confidence interval] were respectively 1.8 [1.5-2.2] for major depressive episode, 1.7 [1.3-2.2] for general anxiety disorder, and 1.6 [1.2-2.2] for phobia. A significant association between voice disorders and sick leave was also demonstrated (1.5 [1.3-1.7]).

**Conclusion:**

Voice disorders were frequent among French teachers. Associations with psychiatric disorders suggest that a situation may exist which is more complex than simple mechanical failure. Further longitudinal research is needed to clarify the comorbidity between voice and psychological disorders.

## Background

Voice disorders cover a wide range of troubles which could be related to various aetiologies, including organic lesion of vocal folds (acquired or congenital), deficient control of breathing, deficient control of laryngeal articulation, as well as psychological difficulties. As highlighted in a recent review of the literature [1], teachers — with others professional voice users such as auctioneers [2], individuals in telesales [3] and aerobics instructors [4,5] — are at particular risk of developing voice disorders.

To determine the significance of these troubles among teachers, several investigators have evaluated data from either patient populations being investigated for voice problems, or from general populations of workers including teachers. In a study by Fritzell [6], individuals in the teaching profession were shown to represent the leading group of patients affected (16.3% of consultations). Female, nursery class and music teachers appeared to be particularly affected. Overall, teachers were shown to be more susceptible to suffer from aphonia, oedema, polyps and nodules than the other professionals. Consistent with theses results, Roy et al. [7] found that teachers had almost twice as many voice disorders at the time of the questionnaire (11.0% vs. 6.2%), and twice as many during their past history (57.7% vs. 28.8%) than the adults in the remaining general population. For their part, Smith et al. [8] showed that teachers had a 3.5-fold higher odds ratio of developing vocal symptoms (OR = 3.5; 95%CI [2.3-5.4]) than non-teachers.

On the whole, findings concerning cofactors of voice problems are inconclusive. Among them, psychological factors have attracted some attention [9,10]. In teachers particularly, stress and psychological tension could play an important role [11,12], but data on this particular relationship are scattered.

Besides computing descriptive figures to understand the full extent of the voice problem in teachers at the national level, we aimed at adding to the emerging literature on the association between voice disorders and psychological status. In accordance with available preliminary results relating voice complaints to psychological distress and/or psychopathology diagnoses, we hypothesized that teachers who reported voice disorders were more susceptible than the others to suffer from psychiatric disorders such as anxiety or depression. To test our working hypothesis, we took advantage of data on a large sample of policyholders of the health care insurance company of the National Education system. Among many other factors, this epidemiological study asked a number of questions on voice disorders, as well as on mental health.

## Methods

### Study population

The study population comprised nationally insured members (compulsory regimes) of the "Mutuelle Générale de l'Education Nationale" (MGEN), a health care insurance company which manages social security reimbursements of everyone working or who had worked in France within the public education system. 45% of these insured members were retired; concerning profession, 65% were teachers and the remaining 35% were non-teaching staff members in charge of general discipline, researchers, librarians or employees/managers in public services. In January 2005, 20,099 of the MGEN insured members, aged 18 years or over, were selected at random from the MGEN health plan records (sample in the 100th on the basis of the internal number of the affiliated MGEN members, unique and individual). Selected individuals received a questionnaire by post accompanied by a letter explaining that the survey was facultative and that anonymity was ensured through the use of identifying numbers. Between January and June 2005, three mail shots were sent, the two latter to non-respondents specifically. As required by the observational research regulation in France, this study was approved by both the national authorities responsible for protecting privacy and personal data: the "Comité Consultatif sur le Traitement de l'Information en matière de Recherche dans le domaine de la Santé" (CCTIRS) and the "Commission Nationale de l'Informatique et des Libertés" (CNIL). Since the objective of this study was to describe voice disorders in teachers, we only analysed data of respondents who declared to be actually giving classes to students (we notably excluded retired teachers).

### Variables of interest

Two questions were included on voice disorders in the section of the questionnaire dedicated to general health. The first concerned the type of problem experienced: "Couldn't you ever have one of the following symptoms? Hoarse voice/frog in the throat/sore throat/loss of voice", with the following possible responses each time: "always", "often", "rarely", "never". The other question concerned history of vocal training. Both questions are available in additional file 1.

For each of the four types of voice symptom considered, a dichotomous variable was constructed on the basis of at least one response among "always"/"often". A fifth variable, named "voice disorders" was coded dichotomously on the basis of at least one voice symptom variable equalling one. Teacher was defined as suffering from voice disorders or not according to this synthetic information.

Mental health problems were defined using the standardised diagnostic questionnaire "*Composite International Diagnostic Interview Short Form" *[13] in its self-administered form (CIDI-SF). This questionnaire also allowed the construction of diagnostic algorithms according to DSM-IV (*Diagnostic and Statistical manual of Mental disorders*) [14] and ICD-10 (*International Classification of Disorders*) [15] criteria for major depression episode, general anxiety disorder, phobias, as well as post-traumatic stress disorder.

The questionnaire also included the SF-36 (*Rand 36-Item Short Form Health Survey*) in its entirety [16], from which the MH (*5-item Mental Health*) scale was used to estimate the level of psychological distress perceived by respondents: the lower the score, the higher the level of psychological distress.

Concerning absence from work, questions were asked about the frequency, duration and reasons for sick leave (long or short duration). A variable named "sick leave" was coded dichotomously as "at least one absence from work during the previous year" vs. "no absence from work".

### Statistical analysis

Data were analyzed using Stata SE 9 software. The level of significance was set at 5%.

First, the analysis sample was described according to sex, age and grade level taught. Then, prevalence of each type of voice disorders was computed by sex. Tetrachoric correlation coefficients were also used to evaluate the strength of the relationship between the different types of voice disorders. Tetrachoric correlation provides a measure of agreement between two binary variables in estimating what the Pearson correlation would be if binary ratings were made on a continuous scale. Indeed, each complaint involving a specific type of voice disorders, although viewed as discrete here, might still be considered as a continuous gradation of varying levels of symptom intensity (i.e. as a latent trait).

The Chi^2 ^test was used to compare the prevalence of voice disorders according to the different group of subjects: variables of interest included sex (male, female), age (18-25, 26-35, 36-45, 46-55, 56-65), grade level taught (unique class nursery/elementary, nursery, elementary, intermediate, regular secondary, vocational secondary, post-secondary, special education), school subject (music/arts, physical activity/dance, other subjects), smoking status (regular smoker, occasional smoker, non/past smoker) and length of time spent in the teaching profession (≤ 5 years service, > 5 years).

To study associations between voice disorders and mental health, the Wilcoxon-Mann-Whitney test was first used to compare level of psychological distress in teachers with or without voice disorders, with respect to gender. Then, multivariate logistic regression models were carried out to calculate sex- and age-adjusted odds ratios between voice disorders (the outcome of interest) and each 1-year psychopathology diagnosis: major depressive episode, general anxiety disorder, phobia, and post-traumatic disorder. The sex-and age-adjusted association between voice disorders and sick leave was also investigated through a logistic regression model.

## Results

The global response rate of the survey was 53%. Of the 10,288 respondents, 3,940 reported to be currently giving classes to students. Specific response rate for these active teachers was estimated to be around 55%, since their expected number among 20,099 was 7,185 (65% of the 55% selected individuals still in activity). This report only presents the data for those 3,646 teachers who provided complete data on voice disorders and grade level taught. Table 1 describes the analysis sample according to sex, age and grade level taught.

**Table 1:** Distribution of participants by sex, age and type of establishment

	**Male** **(*N *= 1,264)**	**Female** **(*N *= 2,382)**
		
	**Frequency (% males)**	**Frequency (% females)**
Age at the survey		
18-25	22 (1.7)	76 (3.2)
26-35	253 (20.0)	512 (21.5)
36-45	302 (23.9)	709 (29.8)
46-55	427 (33.8)	766 (32.2)
56-65	260 (20.6)	319 (13.4)
		
Teaching levels		
Unique class Nursery/Elementary	10 (0.8)	32 (1.3)
Nursery	21 (1.7)	417 (17.5)
Elementary	228 (18.1)	663 (27.8)
Intermediate	280 (22.1)	538 (22.6)
Regular Secondary	259 (20.5)	299 (12.6)
Vocational Secondary	132 (10.4)	143 (6.0)
Post-secondary	235 (18.6)	175 (7.4)
Special Education	99 (7.8)	115 (4.8)

Table 2 shows the prevalence by sex of each type of voice disorders considered in the auto-questionnaire: the most frequent symptoms reported by the two sexes were sore throat, followed by frog in the throat for men and hoarseness of voice for women. Loss of voice was the least frequent problem reported by both men and women.

**Table 2:** Prevalence of voice symptoms by sex

	**Male** **(*N *= 1,264)**	**Female** **(*N *= 2,382)**	**Prevalence comparison ** across sex: p-value*
			
	***N *(%)**	***N* (%)**	
Type of disorder			

Hoarse voice	127 (10.1)	576 (24.2)	< 0.01
Frog in the throat	154 (12.2)	537 (22.5)	< 0.01
Sore throat	213 (16.9)	777 (32.6)	< 0.01
Loss of voice	40 (3.2)	336 (14.1)	< 0.01
Voice disorders - any type	329 (26.0)	1,190 (50.0)	< 0.01

* Chi^2 ^test

When considering voice disorders as a whole, 50.0% of female teachers reported to suffer always or often from at least one type of voice symptom, as compared to 26.0% of males. This difference was strongly significant with a p-value to the Chi^2 ^test less than 0.01.

A number of active teachers underwent vocal training (13.5%). Those who complained of voice disorders were more likely than those who did not to have ever followed a formation to learn how to pose the voice (16.0% vs. 11.7%, Chi^2 ^test: p < 0.01). Similarly, and in accordance with their higher susceptibility to suffer from voice disorders, women were more likely than men to have undergone vocal training (15.6% vs. 9.7%, Chi^2 ^test: p < 0.01).

Table 3 shows the tetrachoric correlation coefficients obtained between the different types of voice symptoms. On the whole these coefficients were strong (from 0.48 to 0.85). The highest correlations were observed between hoarse voice and loss of voice in both men and women.

**Table 3:** Tetrachoric correlation coefficients between the different types of voice disorders

**Type of disorder**	**Hoarse voice**	**Frog in the throat**	**Sore throat**	**Loss of voice**
Hoarse voice	1	0.65	0.54	0.48
Frog in the throat	*0.78*	1	0.65	0.70
Sore throat	*0.67*	*0.67*	1	0.54
Loss of voice	*0.69*	*0.85*	*0.72*	1

Tetrachoric correlation coefficients computed in males are disposed in italics in the lower left of the table, those computed in females are disposed in the upper right.

**Figure 1:**
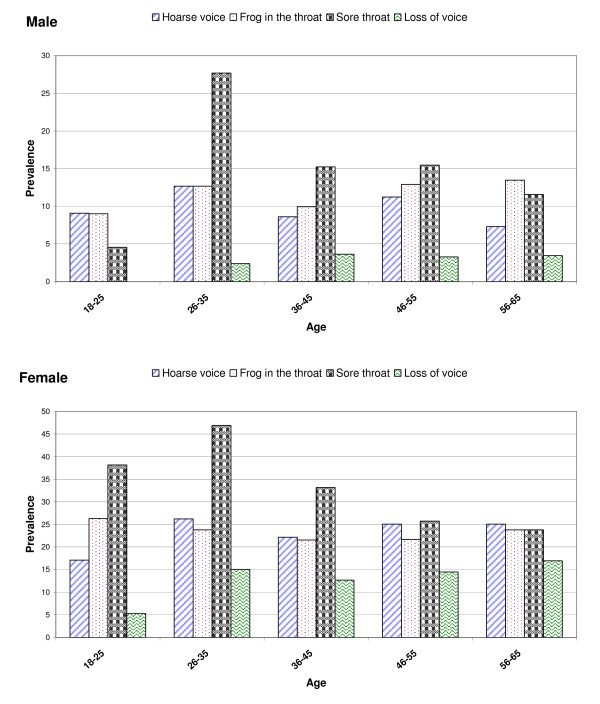
**Prevalence of the different types of voice disorders by age**.

Figure 1 shows the prevalence by sex of the different types of voice symptoms as a function of age. When voice disorders were considered as a whole, a significant non-linear effect was observed across age groups, with both male and female teachers aged 26-35 years being more susceptible to suffer from voice disorders than younger or older teachers (both tests in men and women specific to the 26-35 years class from logistic regression models with voice disorders as outcome: p < 0.01).

There were no statistically significant differences in prevalence of voice disorders between grade levels, despite of a trend towards higher prevalence of voice disorders among teachers of nursery classes than among teachers of other classes. No significant association was found between voice disorders and school subject, or with smoking status. Concerning length of time spent in the teaching profession, women at the start of their careers (≤ 5 years in the profession) more often reported voice disorders than those who had spent longer in the teaching profession (56.2% vs. 49.6%, Chi^2 ^test: p = 0.03).

When using the MH score of SF-36 as an indicator of mental health status, teachers who did not report voice disorders showed lower level of psychological distress than those who did (median MH scores: 68 vs. 60 in women and 72 vs. 68 in men, both Wilcoxon-Mann-Whitney tests: p < 0.01).

Table 4 shows the sex- and age-adjusted OR [95%CI] linking voice disorders to various 1-year psychopathology diagnoses and to sick leave. Results showed a rather strong association between voice disorders and major depressive episode (1.8 [1.5-2.2]), general anxiety disorder (1.7 [1.3-2.2]), and phobia (1.6 [1.2-2.2]), and a weaker association with post-traumatic stress disorder (1.5 [1.0-2.5]). Voice disorders and sick leave were also significantly linked (1.5 [1.3-1.7]). When all psychopathologies were considered simultaneously in a full logistic regression model, associations remained significant only for major depressive episode (1.6 [1.3-2.0]) and general anxiety disorder (1.4 [1.0-1.8]).

**Table 4:** Characterisation of teachers complaining of voice disorders according to mental health and sick leave status

**Voice disorders:**	**No complaint**	**Complaint**	Simple model*		Full model*	
	**(%)**	**(%)**	**OR [95%CI]**	**p-value**	**OR [95%CI]**	**p-value**
Major depressive episode**	10.4	19.0	1.8 [1.5-2.2]	< 0.01	1.6 [1.3-2.0]	< 0.01
Generalized anxiety disorder**	5.6	9.8	1.7 [1.3-2.2]	< 0.01	1.4 [1.0-1.8]	0.04
Phobia**	3.4	5.9	1.6 [1.2-2.2]	< 0.01	1.3 [0.9-1.9]	0.16
Post traumatic stress disorder**	1.6	2.8	1.5 [1.0-2.5]	0.07	1.2 [0.7-2.0]	0.47
						
Sick leave	33.4	46.6	1.5 [1.3-1.7]	< 0.01	—	—

*Logistic regression models were systematically adjusted on sex and age. Simple model: each variable was considered separately; Full model: all mental health diagnoses were introduced simultaneously

**Diagnosis (DSM-IV) referred to the previous one-year

## Discussion

This study, carried out on a large sample of active French teachers, provides some descriptive data on the prevalence of voice disorders in this profession. This condition, which is currently poorly documented in France, merits further investigation. Indeed, our study demonstrated that voice disorders are common among teachers, with one in two female teachers and one in four males complaining of them.

The finding that females were more susceptible to suffer from voice disorders has been consistently reported in previous studies and has certainly to be ascribed to physiological reasons [17,18].

Although it is difficult to establish the prevalence of voice disorders in the general population, Ramig and Verdolini [19] estimated that between 3% and 9% in the United States complained of voice disorders at one time or another. In teachers more specifically, prevalence figures found in our study were higher than those described in other similar studies, ranging from 12% to 26% depending on the sample and the method used [8,20-23]. All of these studies assessed voice disorders in smaller groups of teachers than in ours, and they used various self-administered questionnaires. Of these studies, only one [23] proposed the temporal concept for the prevalence of voice disorders. In this study, 16% of teachers reported having voice disorders at the time of the study, 20% reported having had problems during the previous academic year and 19% reported having had problems during the course of their teaching career. Since then, De Jong et al. [24] demonstrated in a group of 1,878 Dutch teachers that more than half of them reported voice problems during their career and about one fifth had a history of sick leave due to voice problems. The authors also reported than more than 12% of the teachers had experienced voice problems during their training and this group reported significantly more voice complaints and sick leave due to voice problems in their career than the colleagues without voice problems during the training.

We were not able to demonstrate a significant difference in voice complaints according to grade level or school subject taught. Yet, voice disorders in relation to the subjects taught have been investigated by a number of authors. In particular, Thibeault et al. [25] concluded that the subject taught could be an important contributing factor to voice disorders, with teachers of chemical sciences, of music and singing, and of drama being at particular risk.

We have found that women at the start of their career were more susceptible to suffer from voice disorders than those who have been in the job for longer. This result, surprising at first glance given the generally accepted idea of a vocal performance decreasing with age, is in fact consistent with available literature: in a sample of 1,875 teachers of primary and secondary education, Kooijman and al. [26] observed a significant decrease of voice complaints during the career. Similarly, Simberg et al. [27] demonstrated that novice teachers were more subject to vocal symptoms. These authors reported that 34% of novice teachers (n = 226) complained of two (or more) vocal symptoms from the first month of teaching. After one year of teaching, this figure stabilised at 20%. This decreasing prevalence of symptoms may be attributed to coping strategies or a greater tolerance of the vocal problem, but also especially to more teaching experience, which may bring to decrease the need to speak laughter.

In France, voice disorders have not been clearly identified as a professional disease, but they have started to be taken into account during teachers' training. In addition of public health motivations, our study provides economic arguments to prevention requirements in showing a strong association between the presence of at least one voice disorder and the report of at least one absence from work during the previous year. In accordance with this fact, Titze et al. [28] noted that 20% of American teachers monitored for voice disorders reported missing between 1 day and 1 week per year of employment because of their vocal state. In the United States as well, Smith et al. [8] reported that more than 20% of teachers (none in the control group) who complained of voice disorders said that they had taken time off work because of this problem. Likewise, for a population of more than 3 millions teachers in the United States, the prevalence of teachers absent for 1 day per year because of voice disorders is estimated to be 18.3%. As a comparison, Russel et al. [23] estimated that 37.8% of teachers were absent for at least 1 day during the previous year because of voice disorders, whereas Urrutikoetxea et al. [29] reported that 17% of teachers were absent from work at one time or another because of their voice. Given the fact that teachers represent a non-negligible portion of the whole working population (2.7% in France), available data united to point out the important financial implications of voice disorders as an important cause of sick-leave.

One interesting feature of the present study is that it highlighted comorbidity between voice disorders and commune mental health troubles, such as major depressive episode and general anxiety disorder. In a previous study, Mirza et al. [10] showed that it is difficult to know which problem is the consequence of the other. They reported that the prevalence of major mental health problems (evaluated using the BSI; *Brief Symptom Inventory*) varied from 7.1-63.6% depending on the type of voice disorder; however their study population was limited to only 47 patients. In another study, White et al. [9] observed using the GHQ (*General Health Questionnaire*) that women with dysphonia presented higher level of psychological distress than the control population; however, once again, there was a low number of subject (51 cases vs. 42 controls). In the present study, we analysed data from a much larger group using recognised, validated psychiatric diagnostic tools. Although strongly statistically significant, the difference in psychological distress level between those who complained of voice disorders and those who did not remains rather slight, as a 4-point difference in the MH-score corresponds to a single substitution between two contiguous modalities in the response of one or the other item among the five of the MH-questionnaire. However, the robust association between voice disorders and diagnoses of main mental health disorders such as major depressive episode and general anxiety disorder definitely supports that voice disorders may occur in a more complex context than simple mechanical failure. As a consequence, special attention has to be paid to teachers with voice complaints. Potential coexistent psychopathologies have to be recognized to avoid misdiagnosis, but also treatment delay.

There are few national publications concerning voice disorders either in the area of ENT or phoniatrics, or in the fields of occupational health, public health, or preventative medicine. In that context, our results are important to allow better management and prevention among teachers confronted with these problems. However, some points remain problematic, notably the definition of voice disorders, as well as the identification of risk factors, or situations favouring their occurrence. The non-optimal response rate of the present study is another limitation, with possibility of bias in prevalence estimates. Data on voice disorders were not available from non-responders, raising the concern that persons with voice disorders were either over- or under-represented in the sample. The study sample shows similar distributions according to sex and grade level taught to those observed at the national level of teachers within the public education system (2005 statistics provided by the National Education Ministry), but younger teachers (less than 30 years old) were slightly under-represented. Given the fact that 26-35 years old teachers were shown to be more likely to complain of voice, prevalence may be underestimated.

When analysing associations between voice and psychological disorders, we have not taken into account possible other confounding factors than sex and age, the objective being a preliminary description of the vocal-psychological comorbidity among this large sample of teachers. As cross-sectional data does not allow causes and consequences to be distinguished, longitudinal research is needed to identify determining factors of voice disorders. A follow-up questionnaire in the present sample of teachers is planned for 2010, and the data collected will be of interest in that context. In particular, longitudinal analysis will enable evaluating the potential impact of certain measures aimed at preventing voice disorders in teachers. Currently, few studies have tried to evaluate the effectiveness of vocal education programs, particularly in the long term. Bovo et al. [30] studied effectiveness of a course on voice care in a group of primary school female teachers through clinical and instrumental evaluation. At 3 months evaluation, participants demonstrated amelioration in the global dysphonia rates, supporting the interest of voice care programs for both future teachers and for those already practicing. In our cross-sectional data, association between voice disorders and vocal training history can not be interpreted in terms of causality. Nonetheless, our results have implications in public health: our findings on comorbidity between voice and mental health disorders support recommendation for awareness and assessment of psychological distress in teachers complaining of difficulties involving vocal skills.

## Conclusion

Voice disorders are frequent among teachers, in particular in women and in teachers at the start of their careers. Such high prevalence of voice complaints advocate for vocal education programs for both student teachers and active teachers.

The association of voice disorders with mental health troubles confirms that a situation may exist which is more complex than simple mechanical failure. Our finding warrants further interdisciplinary research, as a more complete understanding of otolaryngologic and psychiatric interactions is crucial for the efficient management of these conditions.

## Competing interests

The authors declare that they have no competing interests.

## Authors' contributions

EN and MNV contributed equally to this work. EN designed the study. EN and FG performed the statistic analyses. EN drafted the manuscript. VK supervised the study. MNV helped to draft the manuscript and critically reviewed the manuscript and the statistic analyses. All authors read and approved the final version of the manuscript.

## Pre-publication history

The pre-publication history for this paper can be accessed here:



## Supplementary Material

Additional file 1 **Questions on voice disorders**. The data provided are the items investigating voice disorders in the 2005 MGEN health survey auto-questionnaire. Click here for file

## References

[B1] Williams NR (2003). Occupational groups at risk of voice disorders: a review of the literature. Occup Med (Lond).

[B2] McHenry MA, Carlson HK (2004). The vocal health of auctioneers. Logoped Phoniatr Vocol.

[B3] Jones K, Sigmon J, Hock L, Nelson E, Sullivan M, Ogren F (2002). Prevalence and risk factors for voice problems among telemarketers. Arch Otolaryngol Head Neck Surg.

[B4] Heidel SE, Torgerson JK (1993). Vocal problems among aerobic instructors and aerobic participants. J Commun Disord.

[B5] Long J, Williford HN, Olson MS, Wolfe V (1998). Voice problems and risk factors among aerobics instructors. Journal of Voice.

[B6] Fritzell BR (1996). Voice disorders and occupations. Logopedics Phoniatrics Vocology.

[B7] Roy N, Merrill RM, Thibeault S, Gray SD, Smith EM (2004). Voice disorders in teachers and the general population: effects on work performance, attendance, and future career choices. J Speech Lang Hear Res.

[B8] Smith E, Gray SD, Dove H, Kirchner L, Heras H (1997). Frequency and effects of teachers' voice problems. Journal of Voice.

[B9] White A, Deary IJ, Wilson JA (1997). Psychiatric disturbance and personality traits in dysphonic patients. Eur J Disord Commun.

[B10] Mirza N, Ruiz C, Baum ED, Staab JP (2003). The prevalence of major psychiatric pathologies in patients with voice disorders. Ear Nose Throat J.

[B11] Cooper M (1970). Vocal Suicide in Teachers. Peabody Journal of Education.

[B12] Calas M, Verhulst J, Lecoq M, Dalleas B, Seilhean M (1989). [Vocal pathology of teachers] La Phatologie Vocale chez L'Enseignant. Rev Laryngol Otol Rhinol Bord.

[B13] Robins LN, Wing J, Wittchen HU, Helzer JE, Babor TF, Burke J, Farmer A, Jablenski A, Pickens R, Regier DA (1988). The Composite International Diagnostic Interview. An epidemiologic Instrument suitable for use in conjunction with different diagnostic systems and in different cultures. Arch Gen Psychiatry.

[B14] American Psychiatric Association (1994). Diagnostic and Statistical Manual of Mental Disorders DSM-IV.

[B15] OMS (1994). CIM-10/ICD-10 Classification internationale des troubles mentaux et des troubles du comportement Critères diagnostiques pour la recherche.

[B16] Leplège A, Coste J, Ecosse E, Pouchot J, Perneger T (2001). Le questionnaire MOS SF-36: Manuel de l'utilisateur et guide d'interprétation des scores.

[B17] Kawase N, Sawashima M, Hirose H, Ushijima T (1982). A statistical study of vocal cord nodule, vocal cord polyp and polypoid vocal cord, with special reference to the physical and social histories of patients. Ann Res Instit Logoped Phoniatr Tokyo Univ.

[B18] Dejonckere PH, Dejonckere PH (2001). Gender differences in the prevalence of occupational voice disorders. Occupational Voice: Care and Cure.

[B19] Ramig LO, Verdolini K (1998). Treatment efficacy: voice disorders. J Speech Lang Hear Res.

[B20] Pekkarinen E, Himberg L, Pentti J (1992). Prevalence of vocal symptoms among teachers compared with nurses: A questionnaire study. Logopedics Phoniatrics Vocology.

[B21] Sapir S, Keidar A, Mathers-Schmidt B (1993). Vocal attrition in teachers: survey findings. Eur J Disord Commun.

[B22] Smith E, Kirchner HL, Taylor M, Hoffman H, Lemke JH (1998). Voice problems among teachers: Differences by gender and teaching characteristics. Journal of Voice.

[B23] Russell A, Oates J, Greenwood KM (1998). Prevalence of voice problems in teachers. Journal of Voice.

[B24] de Jong FI, Kooijman PG, Thomas G, Huinck WJ, Graamans K, Schutte HK (2006). Epidemiology of voice problems in Dutch teachers. Folia Phoniatr Logop.

[B25] Thibeault SL, Merrill RM, Roy N, Gray SD, Smith EM (2004). Occupational risk factors associated with voice disorders among teachers. Ann Epidemiol.

[B26] Kooijman PGC, Thomas G, Graamans K, de Jong FICRS (2007). Psychosocial Impact of the Teacher's Voice Throughout the Career. Journal of Voice.

[B27] Simberg S, Laine A, Sala E, Rönnemaa A-M (2000). Prevalence of voice disorders among future teachers. Journal of Voice.

[B28] Titze IR, Lemke J, Montequin D (1997). Populations in the U.S. workforce who rely on voice as a primary tool of trade: a preliminary report. Journal of Voice.

[B29] Urrutikoetxea A, Ispizua A, Matellanes F (1995). [Vocal pathology in teachers: a videolaryngostroboscopic study in 1046 teachers]. Rev Laryngol Otol Rhinol (Bord).

[B30] Bovo R, Galceran M, Petruccelli J, Hatzopoulos S (2007). Vocal Problems Among Teachers: Evaluation of a Preventive Voice Program. Journal of Voice.

