# Supplementary material for: Voice disorders and mental health in teachers: a cross-sectional nationwide study
Source: BMC Public Health. 2009 Oct 2;9:370. doi: 10.1186/1471-2458-9-370 (PMC2762990; doi:10.1186/1471-2458-9-370)
Supplement: Additional file 1 — Questions on voice disorders. The data provided are the items investigating voice disorders in the 2005 MGEN health survey auto-questionnaire. [file 1471-2458-9-370-S1.DOC]

1. Couldn’t you ever have one of the following symptoms? (Check the answer of your choice)

|  | Always | Often | Rarely | Never |
| --- | --- | --- | --- | --- |
| - Hoarse voice | ⁪ | ⁪ | ⁪ | ⁪ |
| - Frog in the throat | ⁪ | ⁪ | ⁪ | ⁪ |
| - Sore throat | ⁪ | ⁪ | ⁪ | ⁪ |
| - Loss of voice | ⁪ | ⁪ | ⁪ | ⁪ |

2. Have you ever followed a formation to learn how to pose the voice?

Yes ⁪ No ⁪ Doesn’t know ⁪
